# Motivational State and Reward Content Determine Choice Behavior under Risk in Mice

**DOI:** 10.1371/journal.pone.0025342

**Published:** 2011-09-22

**Authors:** Mona Leblond, David Fan, Julia K. Brynildsen, Henry H. Yin

**Affiliations:** Department of Psychology and Neuroscience, Department of Neurobiology, Center for Cognitive Neuroscience, Duke University, Durham, North Carolina, United States of America; University of Chicago, United States of America

## Abstract

Risk is a ubiquitous feature of the environment for most organisms, who must often choose between a small and certain reward and a larger but less certain reward. To study choice behavior under risk in a genetically well characterized species, we trained mice (C57BL/6) on a discrete trial, concurrent-choice task in which they must choose between two levers. Pressing one lever (safe choice) is always followed by a small reward. Pressing the other lever (risky choice) is followed by a larger reward, but only on some of the trials. The overall payoff is the same on both levers. When mice were not food deprived, they were indifferent to risk, choosing both levers with equal probability regardless of the level of risk. In contrast, following food or water deprivation, mice earning 10% sucrose solution were risk-averse, though the addition of alcohol to the sucrose solution dose-dependently reduced risk aversion, even before the mice became intoxicated. Our results falsify the budget rule in optimal foraging theory often used to explain behavior under risk. Instead, they suggest that the overall demand or desired amount for a particular reward determines risk preference. Changes in motivational state or reward identity affect risk preference by changing demand. Any manipulation that increases the demand for a reward also increases risk aversion, by selectively increasing the frequency of safe choices without affecting frequency of risky choices.

## Introduction

Organisms must often choose between a small certain reward and a larger but less certain reward. Such choice behavior is often called decision making under risk. Risk, in this sense, can be defined as the variance in the desired outcome [1], [2]. For example, one can choose between a certain option of 100 dollars and 50% chance of 200 dollars. For the probabilistic option, it is impossible to predict the exact outcome each time. In the long run, the payoff is the same; only the outcome varies for the risky option. As outcomes are often variable in nature, risk is a common feature of the interaction between organisms and their environments. There is overwhelming evidence that animals (including insects, fish, birds, and mammals) are sensitive to risk in this sense [2].

Elucidating the mechanisms underlying decision making under risk has significant implications for various conditions such as gambling and addiction. Recent studies have begun to examine the neural substrates underlying decision making under risk [3], [4], [5], [6], [7], [8]. Yet, despite an extensive literature, there remains considerable controversy on even the most basic observations and on the conditions that determine risk preference. For example, many studies have found that animals are risk averse, but some have found either indifference or risk seeking [2], [6], [9], [10], [11].

To elucidate the factors that determine risk sensitivity and preference, here we developed a mouse model of risk-seeking behavior using a choice operant task. The advantages of the widely available genetic tools for the visualization, manipulation, and analysis of the mouse nervous system are well known, but to take advantage of these tools in the study of decision making requires a much more thorough understanding of mouse behavior and working models that can generate testable predictions. Although recent studies have used mice to study operant conditioning [12], [13], [14], [15], [16], [17], [18], few have focused on choice behavior, and none so far on decision making under risk.

**Figure 1:**
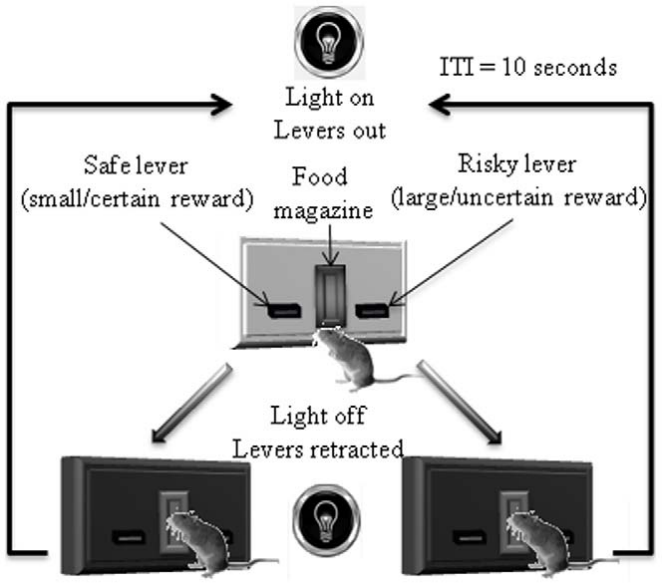
Illustration of the behavioral task. Illumination of the chamber and insertion of both levers signaled the start of the trial. Choosing the safe lever always resulted in an immediate and constant reinforcement (0.01 ml). Choosing the risky lever resulted in a more variable outcome, while maintaining the same overall payoff (e.g. 50% chance of 0.02 ml). Following each choice, both levers were retracted and the light was turned off. The next trial started 10 seconds later (inter-trial interval, ITI).

In this study, we examined the conditions for sensitivity to risk, by measuring the impact of motivational state and reward content on risk preference. We developed a discrete-trial operant choice task to measure risk preference. In this task, two choices yield the same overall payoff. One, however, is always followed by a small reward, whereas the other is only followed by reward probabilistically (**Fig. 1**). We manipulated the level of risk by increasing the variance in the reward outcome while keeping the overall rate of reward constant for the two levers.

We first examined the risk preference of mice under different motivational states, by comparing their choice behavior after food deprivation, water deprivation, or no deprivation (free access to food and water). We also manipulated the content of reward by adding different amounts of alcohol to the sucrose solution. Our results demonstrate that motivational state is a key determinant of risk preference: mice are risk averse when deprived, but indifferent to risk when they are not deprived. In addition, the content of rewards also determines risk sensitivity: with sucrose rewards, mice are highly risk averse, but the addition of alcohol dose-dependently reduced risk aversion. We further show that motivational state and reward identity influence choice behavior by changing the overall demand for reward.

## Results

**Figure 2:**
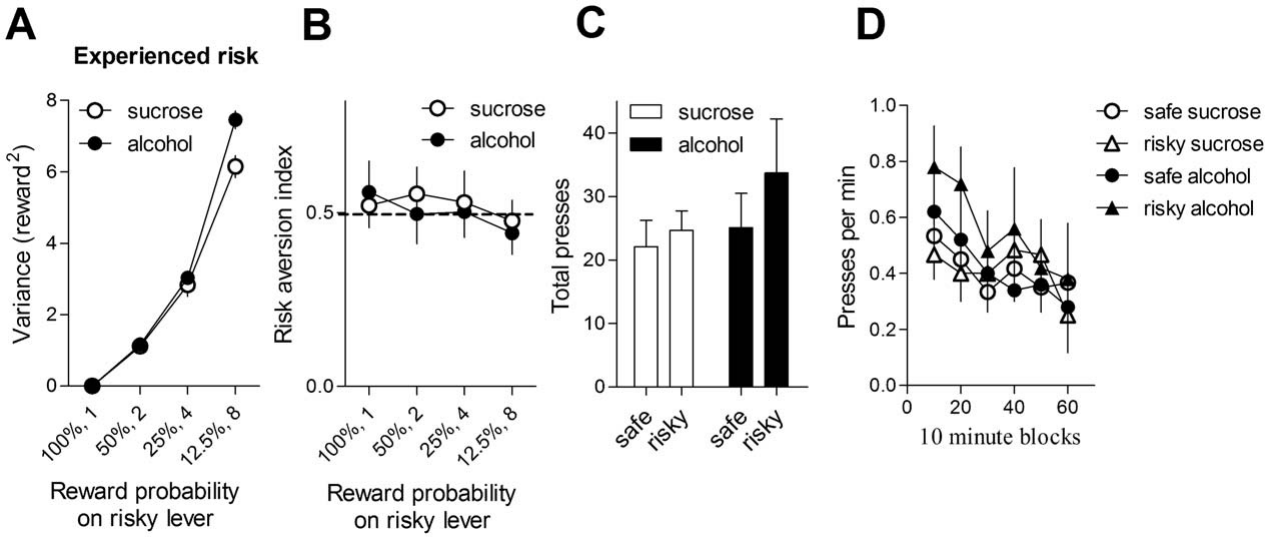
With free access to food and water, mice were indifferent to risk. A. Experienced risk measured by reward variance. The actual experienced risk is calculated by counting the safe reward size (0.01 ml) as 1. When the trial is unrewarded, the outcome is 0, when the outcome is 0.02 ml or twice the safe reward size, the outcome is counted as 2. Error bars indicate standard error of the means (SEM). Sucrose = 10% sucrose; alcohol = 10% sucrose and 20% ethanol. B. Risk preference under different levels of risk (average of last 3 sessions; probability of reward on the risky lever = 12.5%). Mice were trained successively on 4 levels of risk (100%, 0.01 ml; 50%, 0.02 ml; 25%, 0.04 ml; 12.5%, 0.08 ml). Risk aversion index was calculated by dividing number of safe choices by the total number of presses. If the index is greater than 0.5, the animal is risk averse; if it is less than 0.5, the animal is risk prone. C. Number of presses on the two levers (average of last 3 sessions; probability of reward on the risky lever = 12.5%). D. Rate of lever pressing during the last session.

**Fig. 1** illustrates the behavioral task. Experienced risk is the proper measure of risk. For example, if the animal never chose the risky lever at all, there would not be any experienced risk, regardless of the scheduled probability of reward on the lever. Likewise, if the animal only chose the risky lever a few times, and by chance was rewarded every single time, the experienced risk would be low as well. Experienced risk or reward variance (**Fig. 2A**) is calculated as follows. The smallest reward size (0.01 ml) delivered following choice of the safe lever is counted as one reward. When the trial is unrewarded, the outcome is counted as 0; when the outcome is 0.02 ml or twice the safe reward size, the outcome is counted as 2. The variance of the outcomes of all trials from a session was then calculated. Reducing the probability of reward on the risky lever while holding the overall average payoff constant on the two levers dramatically increased the experienced reward variance on the risky lever (main effect of risk level, F_3, 27_ = 381, p<0.001). For this analysis, as well for the analyses below, the average data from the last 3 sessions were used unless otherwise indicated.

### No deprivation

The risk index was calculated by dividing the number of safe choices by the total number of choices. **As shown in** **Fig. 2B**, using a 2-way ANOVA with risk level (12.5%, 25%, 50%, 100%) and reward type (alcohol or sucrose) as factors, we found no interaction between risk level and reward type (F_3, 27_<1, No main effect of reward, F_1, 27_<1, or of risk: F_3, 27_ = 1.38, p>0.05). Neither group showed any sensitivity to the level of risk. The number of lever presses and rate of pressing during the course of the session are displayed in **Fig. 2C and 2D**.

### Water deprivation

**Figure 3:**
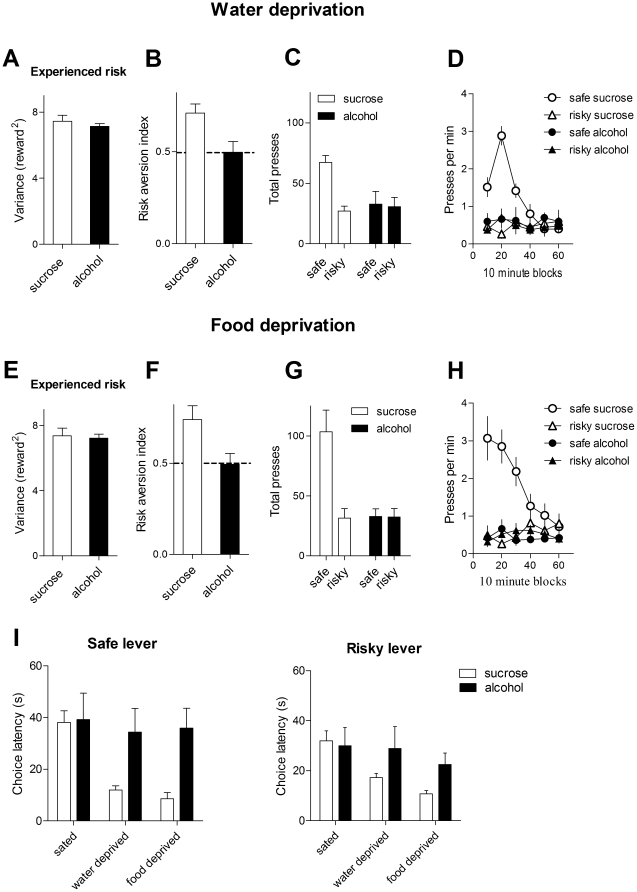
Effects of motivational state and reward content on risk preference. A. Experienced reward variance on the risky lever after water deprivation (average of last 3 sessions; probability of reward on the risky lever = 12.5%). Error bars indicate standard error of the means (SEM). Sucrose = 10% sucrose; alcohol = 10% sucrose and 20% ethanol. B. Risk preference after water deprivation. The sucrose group displayed higher risk aversion, whereas the alcohol group was risk neutral. C. Number of presses on the two levers (average of last 3 sessions). .D. Rate of lever pressing during the last session. E. Experienced reward variance on the risky lever after food deprivation (12.5%). F. Risk preference after food deprivation. The sucrose group displayed higher risk aversion, whereas the alcohol group was risk neutral. G. Total number of lever presses in a session (average of last 3 sessions). H. Rate of lever pressing during the last session (12.5%). I. Choice latency (the time it takes the animal to press the lever once the trial starts). After either food or water deprivation, sucrose group showed much shorter latency compared with the alcohol group (12.5%, average of last 3 sessions).

There is no significant difference between sucrose and alcohol groups in experienced risk (**Fig. 3A**, unpaired t test, p>0.05), but the sucrose group is much more risk averse than the alcohol group (**Fig. 3B**, unpaired t test on the risk aversion index, p<0.05). As shown in **Fig. 3C**, there is a significant interaction between reward type and lever (F_1, 9_  = 7.1, p<0.05), a main effect of reward (F_1, 9_ = 5.0, p = 0.05), and of lever: F_1, 9_  = 8.6, p<0.05). Post hoc tests revealed that the sucrose group chose the safe lever more often (p<0.05), whereas the alcohol group did not (p>0.05). The presses per minute during the course of the session are shown in **Fig. 3D**. Clearly, during the 1 hr session, mice in the sucrose group initially preferred the safe lever, but this strong preference is reduced gradually, so that after roughly 40 minutes there was no longer any preference for the safe lever. Choice of the risky lever remain constant. By contrast, in the alcohol group there was never a significant difference between the safe and risky choices at any point during the session.

### Food deprivation

There is no significant difference in experienced risk between sucrose and alcohol groups (**Fig. 3E** **,** unpaired t test. p>0.05). The risk index is significantly higher in the sucrose group compared with the alcohol group (**Fig. 3F**, unpaired t test, p<0.05). **Fig. 3G** shows total number of presses. There was a significant interaction between reward type and lever (F_1, 9_ = 7.2, p<0.05), a main effect of reward (F_1, 9_ = 12.9, p<0.05), and a main effect of lever (F_1, 9_ = 7.4. p<0.05). The sucrose group chose the safe lever more often (p<0.01), whereas the alcohol group did not (p>0.05.). **Fig. 3H** shows lever presses per minute during the session. The pattern is highly similar to that in **Fig. 3D**.

Another informative measure is choice latency, the time it takes the animal to press the lever once it is inserted. On the safe lever, clearly the overall choice latency is significantly higher when the mice were deprived (**Fig. 3I**). A 2-way ANOVA with reward type and motivational state as factors revealed a significant interaction between them (F_2, 18_ = 6.5, p<0.01), a significant effect of reward type (F_1, 18_ = 4.9, p = 0.05), and a significant effect of motivational state (F_2, 18_  = 11.3, p<0.001). In, particular, when sated, there was no difference between the two groups in choice latency (**Fig. 3I**, average values for the last 3 sessions of training, p>0.05). After food or water deprivation, however the choice latency on the safe lever is much lower in the sucrose group compared to the alcohol group (**Fig. 3I**, ps<0.01). On the risky lever, there was no interaction between reward type and motivational state (F_2, 18_ = 3.0, p>0.05), no effect of reward type (F_1, 18_ = 1.4, p>0.05), but a significant effect of motivational state (F_2, 18_ = 10, p<0.01). Overall, for the risky lever the latency was also significantly lower after either food or water deprivation. After food deprivation, latency was also significantly lower in the sucrose group (p<0.01); but there was no significant group difference after water deprivation (p>0.05).

**Figure 4:**
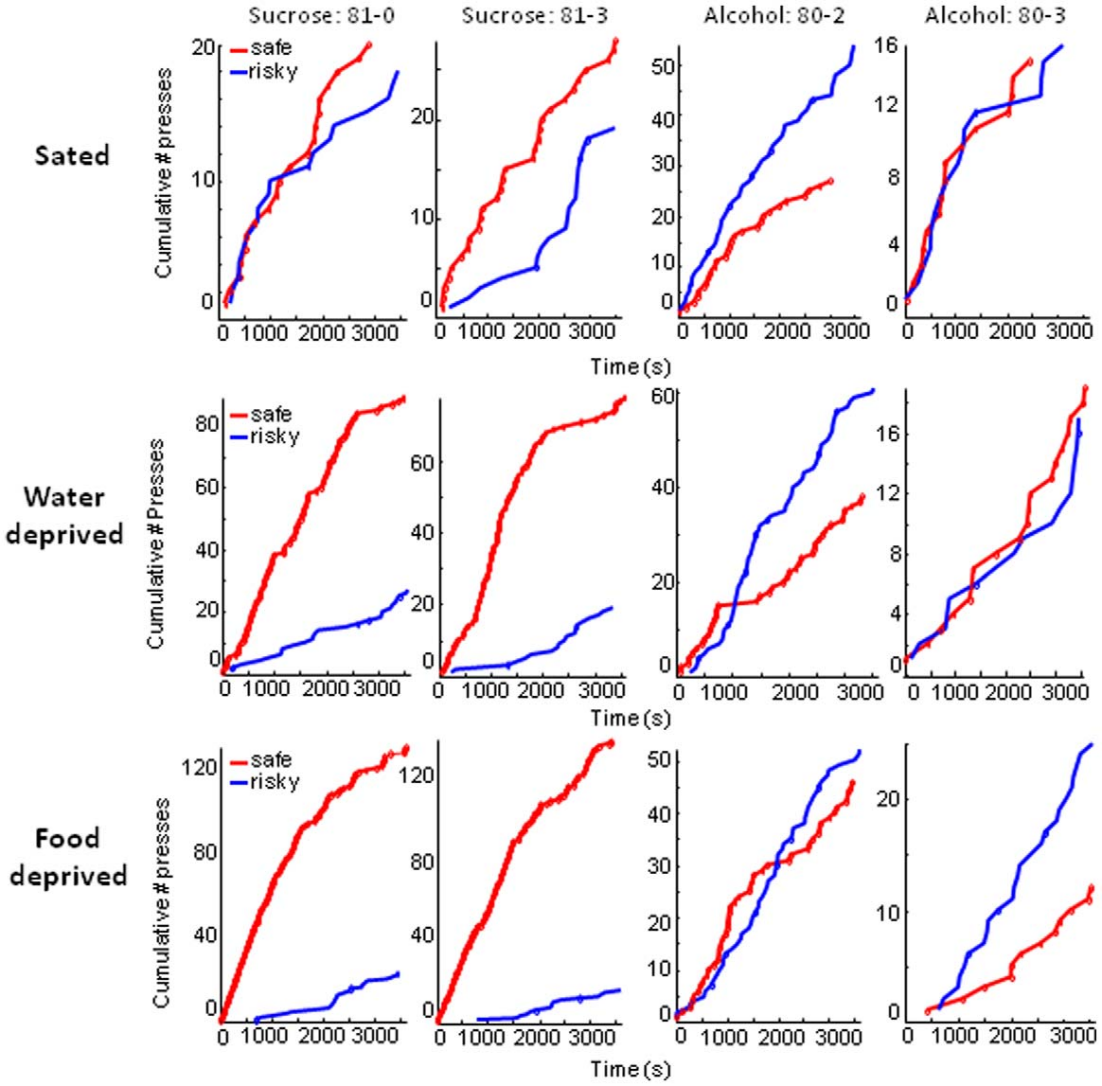
Cumulative records of lever pressing under different conditions. Data from 4 mice are shown from left to right: 2 receiving 10% sucrose solution (sucrose), and 2 receiving 10% sucrose plus 20% alcohol (alcohol). Each graph shows data from the last session (12.5%). The identity of the reward (sucrose = 10% sucrose solution; alcohol = 10% sucrose plus 20% ethanol) and the animal number are shown on top.

As shown in **Fig. 4**, the behavioral data from individual mice agree with our overall summary of the data. The cumulative records from 2 mice from each group clearly demonstrate the robust effects of deprivation and of reward identity on choice behavior. It is important to emphasize that the effects reported here are observed at the individual level.

### Lever reversal

**Figure 5:**
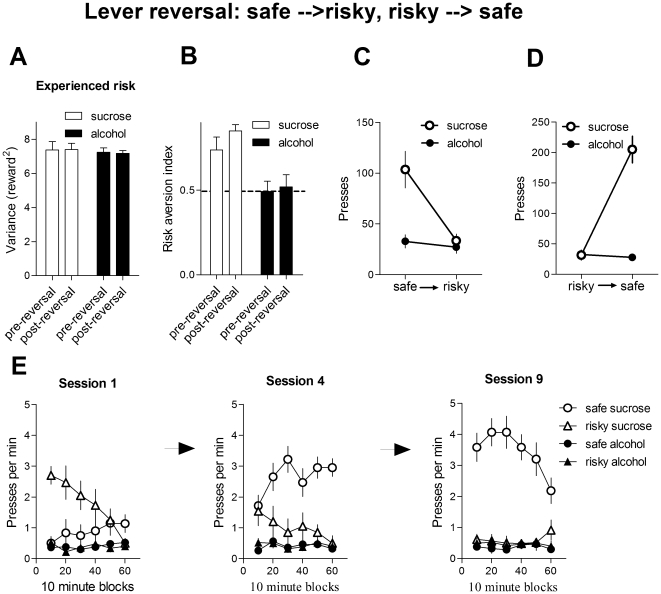
Lever reversal. A. Experienced reward variance on the risky lever (average of last 3 sessions; probability of reward on the risky lever = 12.5%). To control for the lever used, the previously safe lever became the risky lever, and the previously risky lever became the safe lever. Error bars indicate standard error of the means (SEM). Sucrose = 10% sucrose; alcohol = 10% sucrose and 20% ethanol. B. Risk preference after water deprivation (average of last 3 sessions at 12.5%). The sucrose group displayed higher risk aversion, whereas the alcohol group was risk neutral. C. Presses on the left lever: from safe to risky (average of last 3 sessions at 12.5%). The sucrose group decreased pressing on the lever once it switched from safe to risky (100% probability of 1 reward to 12.5% probability of 8 rewards), but the alcohol group did not. D. Presses on the right lever: from risky to safe average of last 3 sessions at 12.5%). The sucrose group increased pressing on the lever once it switched from risky to safe (12.5% probability of 8 rewards to 100% probability of 1 reward), but the alcohol group did not. E. The time course of lever reversal. Rate of lever pressing during the first, fourth, and ninth session.

To control for any lever preference, we reversed the lever, so that the previously safe (left) lever became risky and the previously risky lever became safe. (12.5%, **Fig. 5A**). There was no significant interaction between reward type and lever, no main effect of reward or of reversal (all Fs<1). When we compared risk preference before and after lever reversal (**Fig. 5B**), we found no interaction between lever and reward type, showing that the risk index remained the same after lever reversal. There was a significant main effect of reward type (F_1, 9_ = 26.6, p<0.001), indicating that risk aversion is higher in the sucrose group. There was no effect of lever reversal ( F_1, 9_ = 1, p>0.05) on risk preference. More specifically, a two-way ANOVA revealed a significant interaction between lever and reward (**Fig. 5C**, F_1, 9_ = 9.2, p<0.05), a main effect of reward (F_1, 9_ = 10.4, p<0.05) and of lever (F_1, 9_ = 12.7, p<0.05). Planned comparison revealed that the sucrose group reduced the presses on the left lever once it switched from safe to risky (p<0.05), but the alcohol group did not (p>0.05). For presses on the right lever, a two-way ANOVA with reward and lever revealed a significant interaction between these factors (**Fig. 5D**, F_1, 9_ = 39.6, p<0.05), a main effect of reward (F_1, 9_ = 49, p<0.05), and a main effect of lever (F_1, 9_ = 35.6, p<0.05). Post hoc analysis revealed that the sucrose group increased pressing on the lever once it switched from risky to safe (p<0.05), but the alcohol group did not (p>0.05). The time course of reversal is shown in **Fig. 5E**.

### Reward reversal

**Figure 6:**
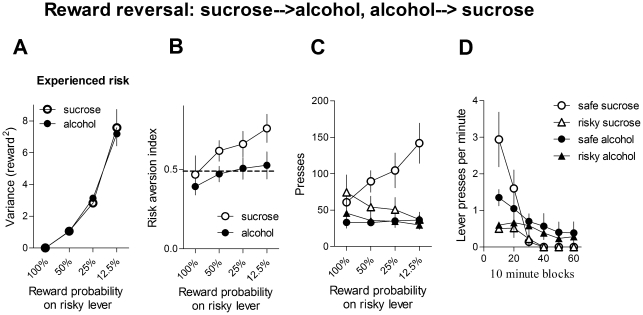
Reward reversal. To demonstrate that the effects we observed were due to the identity of the reward outcome, we also reversed the identity of the reward (sucrose to alcohol and alcohol to sucrose). A. Experienced reward variance on the risky lever average of last 3 sessions at 12.5%). B. Risk preference (average of last 3 sessions at 12.5%). The sucrose group displayed higher risk aversion, whereas the alcohol group was risk neutral. C. Total presses on the two levers (average of last 3 sessions at 12.5%). D. Rate of lever pressing in a session (average of last 3 sessions at 12.5%).

To control for the effect of exposure to specific rewards, we then reversed the reward identity for the two groups, so that the sucrose group received alcohol and the alcohol group received sucrose. We found no significant difference between sucrose and alcohol groups in experienced risk (**Fig. 6A** **).** There was no interaction between reward and risk (F_3, 27_<1), no main effect of reward ( F_3, 27_<1), but there was a significant effect of risk level (F_3, 27_  = 134.2, p<0.0001).

**Fig. 6B** shows the risk aversion index for the different risk levels. Using risk level and reward type as factors, a two-way ANOVA revealed no significant interaction between them (F_3, 27_<1, p>0.05), no effect of reward type (F_1, 27_ = 3, p>0.05), and a significant effect of risk level (F_3, 27_ = 5.7, p<0.01). Thus increasing the risk on the risky lever also increased the risk aversion. **Fig. 6C** shows the total lever presses for the different levels of risk (averaged across the last 3 sessions).

For the highest risk level (12.5%), the sucrose group pressed the safe lever more frequently (p<0.01, planned comparison), whereas the alcohol group pressed both levers equally often (p>0.05, **Fig. 6C**). **Fig. 6D** shows lever pressing during the course of the last session.

**Figure 7:**
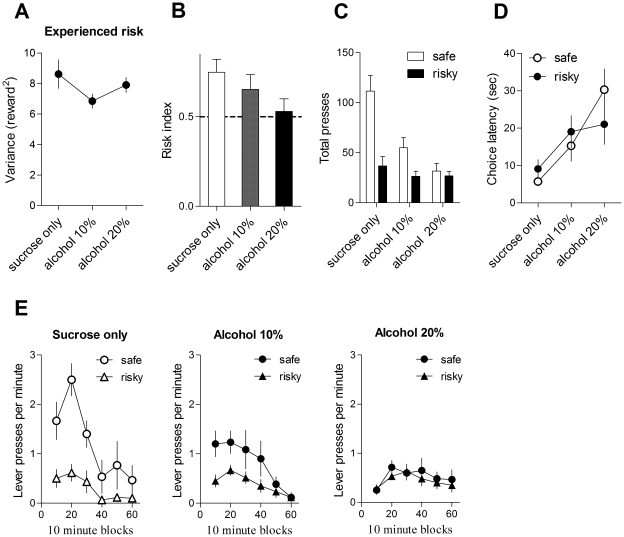
Reducing alcohol concentration reduces risk aversion. A. Experienced reward variance. Error bars indicate standard error of the means (SEM). Sucrose = 10% sucrose; alcohol = 10% sucrose and 20% ethanol. B. Risk preference of mice when the concentration of alcohol was manipulated. Alcohol dose-dependently reduced risk aversion. C. Number of presses. The addition of alcohol to the sucrose reward dose-dependently reduced the number of safe choices. D. Increasing alcohol concentration increased choice latency. E. Rate of lever pressing during the last session.

Using another group of mice, we compared risk preference while manipulating the concentration of alcohol (0%, 10%, and 20% ethanol while maintaining 10% sucrose concentration). There is a main effect of concentration on risk preference (**Fig. 7B**, F_2, 5_ = 6.53, p<0.05). The addition of ethanol dose-dependently reduced risk aversion, largely due to a reduction of pressing on the safe lever (**Fig. 7C**). In addition, alcohol also dose-dependently increased choice latency. Using lever and alcohol concentration as factors, a 2-way ANOVA revealed no interaction between these factors (F_2, 20_ = 2.2, p>0.05), no main effect of lever (F_1, 20_<1), but a main effect of alcohol concentration (**Fig. 7D**, F_2, 20_  = 13.6, p<0.001). Latency on both levers increased as alcohol concentration increased.

## Discussion

We investigated choice behavior in mice using a discrete trial operant procedure, in which the animal must choose between a safe option and a risky option. The risky option yields the same overall payoff as the safe lever, but with higher variance in the outcome. We found that motivational state had a significant impact on risk preference when the reward is sucrose: whereas non-deprived mice were risk neutral, choosing both levers equally (**Fig. 2**), when deprived of either food or water they displayed high sensitivity to the level of risk, showing considerable risk aversion (**Fig. 3** and **Fig. 6**). We also found that the content of the reward is a major determinant of risk preference: the addition of alcohol to the same sucrose solution dose-dependently reduced risk aversion. These novel results shed new light on the mechanisms of choice behavior under risk.

### Motivational state and the budget rule

Our finding that deprivation increases risk aversion is at odds with a popular model of decision making under risk. According to the so-called "budget rule," on a positive energy budget (sated) animals should be risk-averse but on a negative budget (deprived) animals should be risk-prone [2], [19]. The demonstration that deprivation increases risk aversion (**Fig. 3**) falsifies the energy budget hypothesis, if "positive energy budget" is equated with satiety and "negative energy budget" with deprivation. The budget rule takes the perspective of the fictional ideal observer, ignoring the actual perceptual variables that the animal could monitor. According to its underlying assumption, the animal can know in advance that the safe option will not be sufficient for survival, thus preferring to "gamble" instead with the risky option. It is not clear whether such knowledge is ever available to the animal in a natural environment.

More importantly, our results show a simple relationship between motivational state and risk preference, at least when the reward is a 10% sucrose solution: deprivation simply increases risk aversion. In the course of a 1-hr session, mice are initially very risk averse, choosing the safe lever almost exclusively; but as they became sated they gradually reduced choice of the safe lever (e.g. **Fig. 3**). This simple finding may explain why there is widespread disagreement on risk preference [2], [9]. Because previous studies did not usually monitor the motivational state of the animal, it is impossible to establish whether animals are risk seeking or risk prone. Variations in deprivation levels, session length, and reward size can have dramatic effects on risk preference.

The critical question is not *whether*, but *when*, animals are risk averse or risk prone. And our results clearly demonstrate that deprivation results in risk aversion. Yet we did not observe risk seeking behavior. It is important to note that a critical feature of our experimental design is to hold constant the overall payoff for the two choices, so that the long-term payoff is not a confound in determining risk preference [5]. It remains for future studies to determine whether animals can be risk-prone when the overall payoff is the same for the two choices.

### Alcohol and reward content

We found a notable exception to the rule that deprivation increases risk aversion. The other main finding from our study–insensitivity to risk when alcohol is added to the sucrose solution–shows that not only motivational state but also reward content can determine risk preference. Alcohol consumption is commonly believed to increase risk taking and impulsivity, though empirical evidence in support of this claim is lacking. Because the amount of alcohol consumed (∼4 g/kg) in one training session was sufficient to produce intoxication, it is possible that intoxication was directly responsible for indifference to risk. This possibility can be ruled out, however, because mice were already risk neutral at the beginning of the session, before they consumed significant amounts of alcohol (**Fig. 3**). Thus it does not follow that exposure to alcohol increases risk seeking–i.e. that a drunk animal would choose the risky lever more frequently.

Why then did the addition of alcohol to the sucrose solution alter risk preference? Our mice did not simply find the alcoholic solution aversive, thus limiting their intake. They drank as much as possible in one hour, but consider what would happen should the behavior remain the same whether or not the reward contained 20% alcohol. Based on the total amount of sucrose earned during a typical session (∼1.2 ml), had a mouse actually consumed 1.2 ml of 20% alcoholic solution, it would have ingested the equivalent of ∼1 g (∼40 g/kg) of alcohol in one hour, an exceedingly high dose expected to result in alcohol poisoning if not death. It is hardly surprising, then, that the mouse limited the total amount of alcohol consumed. The addition of alcohol simply lowers the demand for the reward, because tolerance is the bottleneck in determining the demand for any alcoholic solution. If so, then diluting the alcoholic solution is expected to increase demand (much as one can drink more wine than whiskey) and restore risk aversion. We tested this prediction in Experiment 2. Indeed, reducing the alcohol concentration from 20% to 10% increased overall demand (**Fig. 7C**); it also produced a significant increase in the number presses on the safe lever without changing choice of the risky lever, resulting in increased risk aversion (**Fig. 7B**).

The observation of insensitivity to risk at higher alcohol concentrations has implications for our understanding of alcohol abuse. The concentration of alcohol is an important determinant of demand for alcoholic beverages. With "stronger" drinks, the seeking behavior can become insensitive to risk, suggesting that alcohol seeking behavior is more likely to persist under risky or partial reinforcement conditions.

### Mechanisms of choice behavior under risk

The influence of deprivation and reward identity on risk preference can be reconciled when the effects of these manipulations on demand is taken into account. Both satiety and the addition of alcohol decrease demand for the reward. More generally, at a given level of risk, increasing demand promotes risk aversion, whereas decreasing demand reduces risk aversion. The results from Experiment 2 provide additional support for this generalization, as the addition of alcohol to the sucrose solution dose-dependently reduces demand and as well as risk aversion. But describing the deprived animals as "risk averse" neglects critical features of the data. Mice press the risky lever less often only when risk is increased significantly. Given the same level of risk, deprivation did not reduce pressing on the risky lever; rather, it increased the number of safe presses (**Fig. 6C**). A more accurate generalization, then, is that increasing demand increases choice of the safe lever, and decreasing demand decreases choice of the safe lever.

In a homeostatic physiological system controlling for food intake, the actual intake is compared with the desired amount, generating an error signal that is translated into action. The reward seeking behavior terminates only when the input somehow matches the desired amount, which is influenced by various factors such as reward identity and motivational state. We can assume, then, that the choice of either safe or risky lever is a result of some error signal specifying how much more is needed to satisfy the current demand, an error signal that can only be reduced by the earned reward. In the absence of the reward input, the error accumulates, because there is no input to cancel the reference signal. Either the safe or risky choice can result in error reduction, but the two choices are associated with different feedback functions. When sated, choice reflects the relative overall yield on the two levers. So long as the overall yield is equal for the two choices, mice are risk neutral, choosing the risky and safe levers equally; the rate of error accumulation is sufficiently slow so that it does not matter whether the animal chooses the fixed or variable reward input. With deprivation, however, the animal could no longer tolerate long periods of no reward imposed by the risky feedback function. To reduce the fast accumulating error, it must choose the safe option more frequently. An analogy may be helpful here: if water is leaking into a boat very slowly, it does not matter whether one gets rid of it with a cup or, taking more time and effort, with a large bucket, if the long-term average amount of water removed by either method is comparable; but if the leak is large with water rushing into the boat, then one is forced to use the cup more frequently.

Although the above account can hardly be considered a genuine model, it at least suggests that such a model is possible without unwarranted assumptions that plague previous theories. And it also makes testable predictions, as the demand for any reward can be measured easily by actual consumption, which is the case in our experiments. This is possible when the feedback function permits the animal to exert full control over the desired amount (i.e. the maximum achievable reward rate is much higher than the rate the animal actually maintains). Any manipulation that changes the demand for a reward, then, is predicted to have the corresponding effects on risk preference. If this generalization holds, it can be the empirical basis for any theory of decision making under risk.

## Materials and Methods

### Ethics Statement

All procedures were approved by the Institutional Animal Care and Use Committee at Duke University and followed National Institutes of Health guidelines (Protocol Number: A062-11-03).

### Subjects

All experiment were carried out in accordance with the Duke University Animal Care and Use Committee Policy. C57BL6/J male mice (∼3 months of age, Jackson Laboratories, Bar Harbor, ME) were used in all experiments.

### Apparatus

Experiments took place in Medical Associates (St. Alban, VT) operant chambers, as described previously [16]. Sucrose and sucrose/alcohol solutions were dispensed by a syringe mounted on a single speed infusion pump. A computer using Med-PC software controlled the chambers.

### Instrumental training

Initial lever-press training consisted of 5 continuous reinforcement (CRF) sessions for the left lever, and 5 CRF sessions for the right lever. In a typical CRF session, the light was on and the respective lever out. A single press resulted in the delivery of 0.01 ml of solution into the food cup. All sessions ended after 120 rewards or after 60 minutes had elapsed.

### Risk task

We developed an operant choice task to study the impact of risk on choice behavior [20]. In this task, two choices yield the same overall payoff. One, however, is always followed by a small reward (0.01 ml of 10% sucrose solution, or 10% sucrose and 20% ethanol solution, or 10% sucrose and 10% ethanol solution), whereas the other is only followed by reward probabilistically (**Fig. 1**). There are 4 different levels of risk associated with the risky lever: 100% chance of 0.01 ml solution, 50% chance of 0.02 ml solution, 25% chance of 0.04 ml solution, 12.5% chance of 0.08 ml solution. The probability distribution and the magnitude of the larger reward were arranged so that its expected value always equaled that of the small and constant reward associated with the safe lever.

At the beginning of each session, the light was turned on and both levers were inserted. Choosing either the left or the right lever ended trial: the light was switched off, the levers were retracted, and the reward was delivered following the scheduled probabilities. The next trial started after an inter-trial interval (ITI) of 10 seconds. All sessions ended after 60 minutes. Mice received one session of training each day.

The mice were first tested with free access to water and food, then water restricted, and finally food restricted (see below). Press reversal, reward reversal, and reward content experiments were all performed when the mice were food restricted.

### Experiment 1: the effect of motivational state on risk sensitivity

Mice were assigned to two groups: "alcohol" (n = 5) and "sucrose" (n = 6). The sucrose group received 10% sucrose solution as reward. The alcohol group received 10% sucrose mixed with 20% ethanol. For the sated condition, mice had free access to water and food in their home cages. For water deprivation, they were allowed one hour of access to water per day, one hour after the completion of the daily training session. For food deprivation, their body weights were monitored daily and kept at about 85% of free feeding weight; they were given 2–3 g of home chow 1 hour after the training session each day.

When sated, mice were trained for 5 days on each of the risk levels (in the following sequence: 100%, 50%, 25%, and 12.5% chance of the reward on the risky lever). After the completion of training for the sated condition, mice were water deprived, and their choice behavior was assessed over 5 days at the highest risk level (12.5%). They were then food deprived and again tested for 5 days at the highest risk level.

#### Lever reversal

To control for the effect of lever position on risk behavior, the risky and safe levers were reversed. Pressing the right lever now resulted in the constant reinforcement, while pressing the left lever now resulted in a large but variable reinforcement (12.5%). Mice were trained for 9 days on the new action-outcome pairing.

#### Reward reversal

Finally, to control for the effect of long-term exposure to either alcohol or sucrose on risk behavior, the identity of the reward was switched for the two groups: the alcohol group received sucrose rewards, and the sucrose group received alcohol rewards. Mice were trained for 5 days on each risk level. Moreover, they were trained under the highest risk level first (12.5%), in a descending order of risk level.

### Experiment 2: The effect of reducing alcohol concentration on risk preference

A new group of mice (n = 6) was used for Experiment 2, which is designed to replicate the finding that the addition of alcohol to the sucrose solution reduces risk aversion. The mice were trained as in Experiment 1, except only the highest level of risk (12.5%) was used. The same mice received 3 different types of rewards: 10% sucrose only, 10% sucrose plus 10% alcohol, and 10% sucrose plus 20% alcohol. Risk preference was measured under each condition until choice behavior became stabilized (3 sessions for sucrose only, 7 sessions for 10% alcohol, and 7 sessions for 20% alcohol).
